# ERP measures of semantic richness: the case of multiple senses

**DOI:** 10.3389/fnhum.2013.00005

**Published:** 2013-01-31

**Authors:** Vanessa Taler, Shanna Kousaie, Rocío López Zunini

**Affiliations:** ^1^School of Psychology, University of OttawaON, Canada; ^2^Bruyère Research InstituteOttawa, ON, Canada

**Keywords:** semantic richness, event-related potentials, N400, metonymy, lexical ambiguity

## Abstract

Semantic richness refers to the amount of semantic information that a lexical item possesses. An important measure of semantic richness is the number of related senses that a word has (e.g., TABLE meaning a piece of furniture, a table of contents, to lay aside for future discussion, etc.). We measured electrophysiological response to lexical items with many and few related senses in monolingual English-speaking young adults. Participants performed lexical decision on each item. Overall, high-sense words elicited shorter response latencies and smaller N400 amplitudes than low-sense words. These results constitute further evidence of the importance of semantic richness in lexical processing, and provide evidence that processing of multiple related senses begins as early as 200 milliseconds after stimulus onset.

## Introduction

When a reader recognizes a written word, information about the word's meaning is activated. Words differ in the amount of meaning-related (semantic) information that they possess, or their *semantic richness*. These differences can be measured in various ways. The first is the number of features generated in feature-listing tasks, where participants are asked to list all the semantic features associated with a given word (e.g., for the item KNIFE, these might include “a utensil,” “is sharp,” “is found in kitchens”). A second measure is the number of associates provided in free association tasks, where participants provide lexical associates for a given word (e.g., BIRD—NEST, BIRD—CAT). A third measure, central in understanding semantic networks, is the number of related meanings—or *senses*—a word possesses. For example, the word TABLE has many related senses (a piece of furniture, a table of contents, to lay aside for future discussion, etc.) while the word GUITAR refers only to a musical instrument.

A growing body of literature indicates that differences in semantic richness lead to different patterns of activation during word recognition. Measures of semantic richness predict response latency in lexical decision, naming, semantic categorization, and self-paced reading tasks (Pexman et al., 2002, 2003, 2008). A functional magnetic resonance imaging (fMRI) study found that semantically rich lexical items elicit less neural activation in left inferior frontal and temporal gyri than words that are more semantically impoverished (Pexman et al., 2007). In combination, these results indicate that increased semantic richness results in more rapid and less effortful lexical processing.

A critical question with respect to semantic richness effects is the timecourse of processing of this information. Event-related potentials (ERPs) are an ideal methodology to investigate this question. ERPs provide a real-time measure of neural processing, with millisecond-level resolution. Several recent studies have used ERPs to elucidate our understanding of the timecourse of activation of information related to semantic richness, including number of features and number of lexical associates (Kounios et al., 2009; Müller et al., 2010; Amsel, 2011; Laszlo and Federmeier, 2011; Rabovsky et al., 2012). In general, the component of interest has been the N400, a negative-going component in the ERP waveform that is traditionally assumed to reflect semantic processing, and whose negativity is inversely related to the semantic expectancy of a word (Kutas and van Petten, 1994). However, the N400 has also been found to be sensitive to context-independent semantic factors (Kounios, 1996) such as concreteness (Kounios and Holcomb, 1994). Semantic priming paradigms have demonstrated that the facilitated processing of a target word that is preceded by a related prime results in a smaller amplitude N400, suggesting that N400 amplitude is negatively related to the ease of processing a word.

With respect to semantic richness, a number of studies have found effects of various types of richness on the ERP waveform. Kounios et al. (2009) found marginally larger amplitude N400s to low- than high-number of feature words; the P2 component, peaking at around 240 milliseconds post-stimulus onset, was also influenced by number of features, with greater amplitude for high- than low-feature words. Rabovsky et al. (2012) investigated two subtypes of semantic richness: number of features and number of associates. Participants performed a lexical decision task on lexical items varying in these two measures. Number of associates did not exert an effect on the ERP waveform, while effects of number of semantic features were observed at central electrodes from 190 to around 500 milliseconds. Like Kounios et al., Rabovsky et al. observed a larger positive peak to low- than high-feature items in the P2/N2 window, arising at around 190 milliseconds post-stimulus onset. High-feature items, however, elicited a larger N400 than low-feature items—in contrast to the findings of Kounios et al. The authors conclude that initial semantic access occurs early and continues to exert an effect on processing at later stages in the reading process, and that partial data transmission occurs between the orthographic and semantic levels prior to completion of orthographic processing (i.e., processing is interactive rather than modular).

A more in-depth examination of semantic feature effects was reported by Amsel (2011), who compared the effect of different semantic feature types on the ERP waveform. Effects were observed prior to 200 milliseconds post-stimulus onset, with different feature types exerting independent effects. Thus, the effects of semantic features are clearly complex, modulated by feature type, and occur very early in the timecourse of lexical processing.

Unlike Rabovsky et al. (2012), other studies have found effects of number of associates on the ERP response. Müller et al. (2010) observed larger N400 components to items with a high number of associates (e.g., SPOON—FORK) than those with fewer associates in a lexical decision task. Laszlo and Federmeier (2011) likewise observed larger N400 amplitudes for items with many associates relative to those with fewer associates. The frequency of the associates also exerted an effect on the ERP waveform: items with higher frequency associates elicited larger N400 amplitudes than those with lower frequency associates.

Both of these studies found an *increase* in N400 amplitude for more or higher-frequency lexical associates, contrary to the prediction that items with more associates (i.e., with richer representations) should be easier to process and thus elicit less neural activation and smaller N400 amplitudes. Laszlo and Federmeier interpreted this finding as indicating that lexical associates, particularly those of high frequency, serve as better “lures” away from the target item. That is, high frequency words generally elicit a smaller amplitude N400 than low frequency words because they are easier to activate; in the case of high frequency associates, there is a greater tendency of the associate to become activated, luring activation from the target item and resulting in larger N400 amplitude. This interpretation is consistent with research suggesting that the N400 reflects inhibitory function (Debruille, 1998); the lexical associate competes with the target item and must be inhibited, leading to a larger N400. Such competition would not be expected in the case of items with multiple related senses, because the senses are not in competition for activation.

The present study extends previous research on ERP measures of semantic richness by examining ERP response to lexical items with high and low numbers of senses, an area that remains to be explored. Our previous research (Taler et al., 2009) found that lexical items with two related literal senses (e.g., CHICKEN meaning animal or meat) elicited smaller N400 amplitudes in healthy older adults than homonyms (i.e., items with two unrelated senses, such as BANK meaning “a financial institution” or “the side of a river”). However, that study did not assess the effect of number of senses, an important measure of semantic richness that has been demonstrated in behavioral studies to exert an effect on lexical processing. Specifically, Rodd et al. (2002) demonstrated facilitated processing for words with multiple related senses and a disadvantage for words with multiple meanings. Consistent with our previous research (Taler et al.), as well as previous behavioral research indicating faster processing of semantically rich items (Pexman et al., 2002, 2003, 2008) including multiple related senses (Rodd et al., 2002), we anticipate that high-sense words will elicit smaller N400 amplitudes and faster response times in a lexical decision task than low-sense words. Unlike lexical associates, related senses do not constitute a “lure” away from the target item, and hence we do not predict an increase in the amplitude of the N400 to high-sense items, as was observed for items with many lexical associates (Laszlo and Federmeier, 2011).

## Methods

### Participants

Participants included 20 right handed native monolingual English speakers (11 females) recruited from the University of Ottawa community. Their mean age was 22 years (*SD* = 1.8) and at the time of testing they had completed an average of 16 years of education (*SD* = 1.1). Prior to the testing session all participants completed a self-report health and history questionnaire to ensure that they were in good health and did not suffer from any conditions and were not taking any medications that are known to affect cognitive function. All participants showed normal cognitive function as measured by the Montreal Cognitive Assessment (MoCA; Nasreddine et al., 2005) and had no neurological or psychiatric history. This study was approved by the Bruyère Research Institute and University of Ottawa research ethics committees.

### Materials and apparatus

#### Stimuli

The experiment included a total of 134 stimuli in three conditions. The “high” condition comprised 32 stimuli with many related senses (e.g., EYE), while the “low” condition comprised 32 stimuli with few related senses (e.g., GYM)^1^. Number of senses was determined using WordNet (Princeton University, 2010). The experiment included 70 pseudowords matched to real word stimuli for length, orthographic neighborhood density (total number of orthographic neighbors, i.e., the N-metric), and bigram frequency by position, using data from the English Lexicon Project (Balota et al., 2002). The stimuli in the two real-word conditions (high and low) were matched for length and for bigram frequency by position using data from the English Lexicon Project, for frequency using log-transformed values from the CELEX database (Baayen et al., 1993), and for familiarity, concreteness, and imageability using data from the MRC Psycholinguistic Database (Coltheart, 1981). They were also balanced for number of lexical associates using the University of South Florida Free Association Norms (Nelson et al., 1998). Current databases do not provide sufficient numbers of items for control of number of features; however, as noted by Rabovsky et al. (2012), number of features and number of associates tend to be highly correlated. High-sense items were of higher orthographic neighborhood density than low-sense items (*p* < 0.05). Stimulus characteristics are provided in Table 1. All stimulus matching was done using independent sample *t*-tests (comparing high to low-sense items, and all real words to pseudowords).

**Table 1:** **Characteristics of experimental stimuli: mean (standard deviation)**.

	**High-sense words**	**Low-sense words**	**Pseudowords**
Number of related senses	6.72 (2.57)	1.70 (0.47)	N/A
Frequency (CELEX log-transformed)	1.32 (0.46)	1.15 (0.55)	N/A
Length	5.22 (1.41)	5.69 (1.35)	5.51 (1.34)
Orthographic neighborhood density	4.81 (4.58)	2.75 (3.34)	3.69 (3.63)
Bigram frequency by position	1340.59 (735.40)	1575.69 (837.66)	1396.66 (688.59)
Concreteness	561.59 (69.30)	571.97 (69.06)	N/A
Familiarity	539.34 (49.55)	530.53 (47.14)	N/A
Imageability	568.13 (52.05)	589.57 (47.69)	N/A
Number of associates	15.09 (5.58)	12.74 (5.40)	N/A

#### Experimental task

Participants completed a lexical decision task, in which they decided if each stimulus was a real word in English or not, and indicated their response using the “a” and “l” keys on the keyboard; electrophysiological recording took place simultaneously. Stimuli were presented in white 18 point Courier New font on a black background for 2000 ms or until a response was detected. Prior to each stimulus, a fixation cross was presented for 500 ms at the center of the monitor and participants were asked to maintain their gaze on the fixation between the presentation of each word. The experiment was run using E-Prime 2.0 presentation software (Psychology Software Tools, Pittsburg, PA, USA). Stimuli were presented to participants on a Dell OptiPlex 780 desktop computer with Windows XP Professional operating system, an Intel Core 2 Duo processor and a 20″ monitor.

#### EEG recording

The continuous EEG was recorded from 32 electrode sites according to the international 10–20 system of electrode placement using tin electrodes and a commercially available nylon cap (Electro-Cap International, INC., Eaton, OH, USA). A cephalic site was used as the ground and all active sites were referenced online to linked ears. Four additional electrodes were used to record the horizontal and vertical electro-oculogram (EOG); the horizontal EOG was recorded from electrodes placed at the outer canthus of each eye and the vertical EOG from electrodes placed above and below the left eye. The EEG was amplified using NeuroScan NuAmps (NeuroScan, El Paso, TX, USA) and was sampled at a rate of 500 Hz in a DC to 100 Hz bandwidth. Electrical impedances were kept below 5 kΩ during EEG recording. The EEG data were processed offline using NeuroScan 4.3 EDIT software (NeuroScan, El Paso, TX, USA). We applied a 30 Hz lowpass filter, vertical EOG artefact was corrected using a spatial filter (NeuroScan EDIT 4.3), and trials containing horizontal EOG artefact exceeding ±50 μV were excluded from averaging, as were trials containing deflections exceeding ±100 μV. The electrophysiological time epoch was 1100 ms comprised of a 100 ms pre-stimulus baseline and 1000 ms following the onset of the stimulus word. Averages were computed based on the three conditions of the experimental task and were baseline corrected to a 0 μV average of the 100 ms pre-stimulus interval. Only correct trials were included in averages and all averages contained a minimum of 30 trials.

### Procedure

Participants were seated in a comfortable chair and informed consent was obtained. Given that this investigation formed part of another study, several neuropsychological tasks were performed prior to setting up the participant for EEG recording. These tasks took approximately 20 min to complete and were followed by the application of the electrodes for EEG recording, which took approximately 30 min. The experimental task examined here took approximately 5 min to complete and was followed by an additional task that was approximately 30 min long. In total the testing session ranged from 1.5 to 2 h with 30–45 min of EEG recording. Following the testing session the purpose of the experiment was explained in detail and any questions that the participant had were answered. Participants were compensated $10 per h of participation.

## Results

All statistical analyses were conducted using PASW Statistics v. 18. Only correct trials were included in the analyses.

### Behavioural results

Trials with RTs exceeding ±2.5 standard deviations from the mean were excluded as outliers, which resulted in the removal of a total of 3.9% of trials from the high sense condition (1.7% errors, 2.2% outliers) and 3.3% of trials from the low sense condition (1.4% errors, 1.9% outliers). The accuracy and reaction time (RT) data were analyzed in separate paired samples *t*-tests. There was an effect of number of senses on RT [*t*_(19)_ = −2.07, *p* = 0.05], but not on accuracy. Specifically, words with many senses elicited faster RTs (*M* = 657.8 ms, *SD* = 137.5) than those with few senses (*M* = 680.0 ms, *SD* = 134.8). In terms of accuracy, both conditions elicited few errors, with an accuracy rate of 98.3% (*SD* = 2.4) for the high sense condition and 98.6% (*SD* = 1.9) for the low sense condition.

### Electrophysiological results

Two participants were excluded from analysis of the electrophysiological data due to technical difficulties during EEG recording; thus 18 participants were included in all analyses of the electrophysiological data. Given that we were interested in the N400, we analyzed sites where the N400 was largest (i.e., central and posterior sites) and only included the time interval that best represented the N400. Visual inspection indicated an early divergence in the waveforms; thus, we examined the 200–550 ms time window by subdividing it into consecutive 50 ms time bins (i.e., 200–250 ms, 250–300 ms, 300–350 ms,…, 500–550 ms). Time was then entered into the ANOVA as a within-subjects variable. We analyzed the midline, and the left and right lateral sites in three separate 2 (Condition: high vs. low) × 3 (Site: C4, CP4, P4 or Cz, CPz, Pz or C3, CP3, P3) × 7 (Time: 0–550 ms) repeated measures analyses of variance where the dependent variable was the average ERP amplitude in each of the 50 ms time bins. First we report the results from the midline sites, followed by the results from the lateral sites.

**Figure 1:**
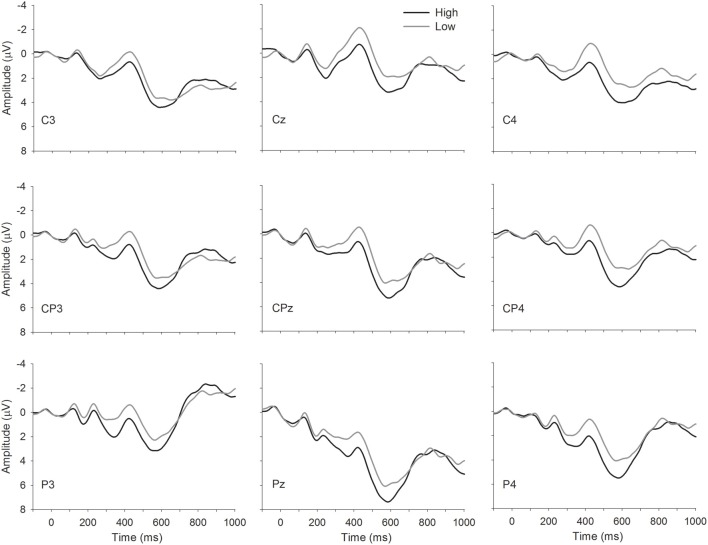
**Grand average waveforms showing high- and low-sense items.** Time is plotted on the *x*-axis and amplitude in microvolts on the *y*-axis; following convention, negative amplitudes are plotted upwards.

Grand averages waveforms are depicted in Figure 1. Analysis of the midline sites revealed a main effect of Condition, *F*_(1, 17)_ = 5.09, *MSE* = 57.35, *p* = 0.04, indicating a larger amplitude N400 for words with few related senses than words with many related senses. This finding was further supported by the analysis of the lateral sites. Right lateral sites demonstrated a similar effect of Condition [*F*_(1, 17)_ = 6.92, *MSE* = 48.20, *p* = 0.02], with words with few related senses demonstrating a larger N400 amplitude than words with many related senses. Finally, analysis of the left lateral sites revealed a trend toward a main effect of Condition, *F*_(1, 17)_ = 3.53, *MSE* = 64.97, *p* = 0.08, again revealing larger N400 amplitudes for words with few related senses relative to words with many related senses. There were no meaningful significant interactions in any of the analyses performed. We note that high-sense items were also of higher orthographic neighborhood density than low-sense items; however, the effect (larger N400 amplitudes for low- than high-sense items) is in the opposite direction to the reported effects of neighborhood density (larger N400 amplitudes for high- than low-neighborhood density items) (Holcomb et al., 2002).

## Discussion

The present study examined behavioral and ERP response to words with high and low levels of semantic richness, as measured by the number of related senses that the word possesses. Participants responded more quickly to high- than low-sense items, consistent with previous research (Rodd et al., 2002; Yap et al., 2012). We found smaller N400 amplitudes to high- than low-sense words in midline and right lateral sites, with the waveforms for the two conditions diverging at around 200 milliseconds post-stimulus onset.

These results indicate that readers activate information about the number of senses a word possesses very early in processing, consistent with previous research on number of semantic features (Amsel, 2011; Rabovsky et al., 2012). However, interestingly, the direction of the effect differs between our findings and Rabovsky et al., who found a larger N400 to high-feature than low-feature items. Similarly, Amsel (2011) found more negativity in the N400 window to high-feature items. Laszlo and Federmeier (2011) also found larger N400s to words with more or higher-frequency lexical associates than fewer or lower-frequency associates. Rabovsky et al. do not offer a theoretical account for their findings, but Laszlo and Federmeier suggest that lexical associates, particularly those of high frequency, may serve as better “lures” for the target item, thus invoking an account similar to that of Debruille (1998), who suggests that the N400 may index inhibitory function. Given that our stimuli differ in the number of related senses and not number of associates, it is not surprising that they do not invoke inhibition. That is, the multiple senses of our stimuli are related and therefore activation of a larger number of senses should facilitate processing because there is no need to inhibit any of the senses, as is the case when an associate is activated. Thus, stimuli with a high number of related senses should elicit faster RTs and smaller amplitude N400s, as the results demonstrate.

These results also contrast with the findings reported in the literature that concrete words elicit larger N400 amplitudes than abstract words (Kounios and Holcomb, 1994; Holcomb et al., 1999; West and Holcomb, 2000), possibly reflecting higher semantic richness associated with high concreteness [although note that Kounios et al. (2009) suggest that previous studies of concreteness may not have adequately controlled for relevant semantic variables]. Similarly, Rabovsky et al. (2012) recently reported effects of newly acquired semantic information: newly-learned words associated with more semantic information showed larger amplitude N400s than those associated with less information.

The findings are, however, consistent with our previous research (Taler et al., 2009), which found smaller N400 components to metonyms (lexically ambiguous items with two related literal senses, such as CHICKEN) than to homonyms (lexically ambiguous items with two unrelated meanings). We argue that metonyms are semantically richer than homonyms, which are associated with two unrelated—and thus competing—lexical entries rather than a single lexical entry or multiple closely-linked entries. This finding indicates that relatedness of senses impacts the ERP response, with reduced N400 amplitude associated with related relative to unrelated senses. Similarly, Rodd et al. (2002) differentiate between related and unrelated senses such that processing is facilitated for words with multiple related senses, but not for words with multiple unrelated senses. Our findings are also in line with behavioral evidence indicating shorter response latencies to high- than low-sense items (Rodd et al., 2002; Yap et al., 2012), as well as with fMRI evidence showing less neural activation to high- than low-number of associate lexical items (Pexman et al., 2007). It should be noted, however, that N400 amplitude does not map perfectly onto response latency; shorter response times to items that elicit larger N400 responses have been reported in the case of orthographic neighborhood density (Holcomb et al., 2002), number of lexical associates (Müller et al., 2010), and concreteness (West and Holcomb, 2000).

In sum, the varied findings reported in the literature indicate that semantic richness effects are far from straightforward. One possibility that arises from this discrepancy is that different measures of semantic richness may exert different influences on lexical processing, a conclusion also reached by Rabovsky et al. (2012). This hypothesis has been explored by Pexman et al. (2008), who found that three measures of semantic richness (number of features, number of associates, and contextual dispersion) accounted for unique variance in a lexical decision task. Similarly, Yap et al. (2012) found that different types of semantic richness exerted different effects in various word recognition tasks, suggesting that various types of semantic information are used adaptively depending upon task demands. We suggest that some types of semantic richness (such as number of lexical associates) may require greater inhibitory control than others (such as number of related senses); this hypothesis is supported by opposite effects on the N400 component of these two semantic factors.

In the present results, we interpret the reduced N400 amplitude to high- relative to low-sense words to reflect reduced processing demands in the former case. This interpretation is supported not only by existing literature (Yap et al., 2012), but also by the shorter response latencies to high-sense words than to low-sense words. The present study focused exclusively on the effect of number of related senses on lexical decision latency and ERP response. Future research should further explore different measures of semantic richness in combination with number of senses, as these measures likely exert differential effects on the ERP response, and may indeed interact. We also note that, although high- and low-sense items did not differ significantly in terms of frequency, high-sense items were numerically higher in frequency than low-sense items, which may have influenced the present results.

In summary, we demonstrated reduced N400 amplitude and shorter response latencies to high-than low-sense lexical items. These results indicate greater ease of processing of words with many related senses relative to words with fewer senses. The ERP waveform began to diverge between the two conditions as early as 200 milliseconds post-stimulus onset, indicating that this semantic information is accessed very early in lexical processing. These results shed further light on the organization of semantic information in the lexicon, as well as the timecourse of lexical access.

### Conflict of interest statement

The authors declare that the research was conducted in the absence of any commercial or financial relationships that could be construed as a potential conflict of interest.

## Footnotes

^1^Six additional real-word items were not included in analyses; participants thus saw 140 stimuli in total.
